# Oleuropein Enhances Stress Resistance and Extends Lifespan via Insulin/IGF-1 and SKN-1/Nrf2 Signaling Pathway in *Caenorhabditis elegans*

**DOI:** 10.3390/antiox10111697

**Published:** 2021-10-27

**Authors:** Shiling Feng, Chunyan Zhang, Tao Chen, Lijun Zhou, Yan Huang, Ming Yuan, Tian Li, Chunbang Ding

**Affiliations:** 1 College of Life Science, Sichuan Agricultural University, Ya’an 625014, China; fsl@sicau.edu.cn (S.F.); zcy18227590979@126.com (C.Z.); chentao293@sicau.edu.cn (T.C.); zhoulijun@sicau.edu.cn (L.Z.); shirley11hy@163.com (Y.H.); yuanming@sicau.edu.cn (M.Y.); 2 College of Agronomy Science, Sichuan Agricultural University, Chengdu 611130, China; lit@sicau.edu.cn

**Keywords:** oleuropein, *Caenorhabditis elegans*, longevity, stress resistance

## Abstract

Oleuropein (OLE) is a secoiridoid glycoside that mainly exists in olives with multifaceted health benefits. The present study aimed to investigate the stress resistance and lifespan extension effects of OLE in *Caenorhabditis elegans*. The results showed that OLE could significantly prolong the lifespan of *C. elegans* by 22.29%. Treatment with OLE also significantly increased the survival rates of worms against lethal heat shock and oxidative stress. Meanwhile, OLE supplementation increased the expression and activity of antioxidant enzymes and suppressed the generation of malondialdehyde in nematodes. In addition, the results from mutants implied that OLE might mediate longevity and stress resistance via DAF-16/FoxO, which played a vital role in the insulin/IGF-1 signaling (IIS) pathway. To further identify the molecular targets of OLE, mRNA level and loss-of-function mutants of IIS-associated genes were investigated. The data revealed that OLE activated IIS by down-regulating the upstream components, *daf-2* and *age-1*. Furthermore, another stress response and longevity pathway in parallel to DAF-16, SKN-1/Nrf2, was also shown to involve in OLE-induced beneficial effects. Collectively, these results provide the theoretical basis that OLE could enhance the stress resistance and increase the lifespan of *C. elegans* through the IIS and SKN-1/Nrf2 signaling pathways.

## 1. Introduction

**Figure 1:**
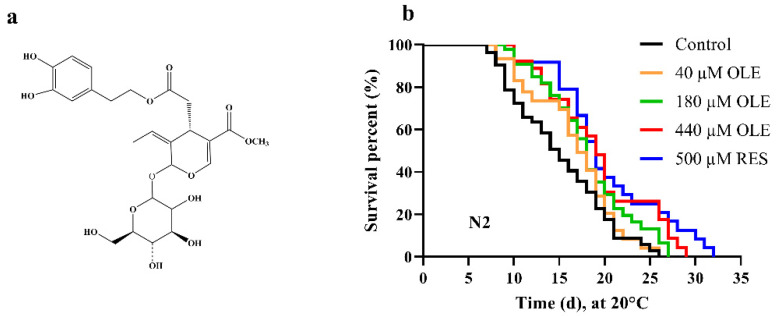
(**a**) Chemical structure of OLE. (**b**) Effect of OLE on the lifespan of wild-type worms.

Oleuropein (OLE), a bitterness substance belonging to secoiridoid, is the major bioactive compound in Oleaceae plants (Figure 1a). This terpene glycoside consists of a molecular of hydroxytyrosol and elenolic acid glucoside linked by a lipase or oleuropein aglycone bond with glucoside by β-glucosidase [1,2]. OLE is distributed in all parts of olive, especially in fruits and leaves. This natural phenolic compound is a desirable component in high-quality olive oil that strongly influences the flavor due to its bitter and pungent sensory notes. Studies showed that OLE and several oleuropein derivates possessed various pharmacological properties of antioxidative, anti-inflammatory, anticancer [3], and delayed age-associated diseases [4,5]. In particular, OLE has been widely reported to exhibit robust antioxidant activity both in vitro and in vivo. Supplementation of OLE protected aged rats model from oxidative stress [6]. Oral administration of 30 mg/kg OLE significantly alleviated arsenic-induced oxidative damage in mice [7]. These beneficial effects of OLE are thought to be directly attributed to its free radicals-scavenging ability, intrinsic redox enzymes, and stress response modulation effects. Resistance to oxidative stress has been extensively correlated with longevity. Although OLE was shown to improve oxidative stress resistance and ameliorates several age-related physiological deteriorations in animal models, the exact effect and underline mechanism by which OLE affects the stress resistance and lifespan has inadequately been characterized.

*Caenorhabditis elegans* is a simple structure and transparent roundworm. This free-living nematode is a powerful genetic model animal mostly known for aging research due to its relatively short lifespan. It has been used by many to evaluate the ameliorative effects associated with longevity and stress of natural products. Studies have uncovered several signaling pathways interfering with stress response and the aging process, such as insulin/insulin-like growth factor signaling (IIS), which was conservative from worms to mammals. In *C. elegans*, IIS originates with insulin-like peptides binding the DAF-2 receptor to activate the phosphoinositide 3-kinase (AGE-1/PI3K). Subsequently, activated AGE-1 triggers the downstream phosphorylation cascade reactions starting from 3-phosphoinositide-dependent kinase 1 (PDK-1) to Akt/protein kinase B family members (AKT-1 and AKT-2), which in turn leads to the phosphorylation of DAF-16/FoxO transcription factor and inhibits its translocalization activity into the nucleus. DAF-16 is a crucial component in IIS that directly regulates longevity and various stress response in worms [8]. Suppressed IIS releases DAF-16 by down-regulating upstream genes, consequently increasing the transcriptional regulation of DAF-16 on downstream targeted genes to extend the lifespan and enhance stress resistance. In addition to DAF-16, some other transcription factors are also regulated by IIS, such as SKN-1, an Nrf2 transcription factor homolog that regulates both longevity and stress response. Part of SKN-1/Nrf2 downstream targets overlaps with DAF-16 targeted genes involved in the process of lifespan extension and stress responses [9].

The present study was aimed to evaluate the stress resistance and lifespan extension effect of OLE using *C. elegans* as in vivo model. For those purposes, the survival rates of worms pretreated with OLE against oxidative stress and heat shock were measured. The lifespan extension effect of OLE was carried out using wild-type N2 worms. The mRNA level of associated genes regulated by OLE was estimated by qRT-PCR. In addition, to further elucidate the mechanism of action, fluorescence and null mutant strain associated with IIS, DAF-16, and SKN-1 pathway was investigated.

## 2. Materials and Methods

### 2.1. Chemicals and Reagents

Oleuropein, resveratrol, paraquat, sodium azide, and DMSO were purchased from Aladdin (Shanghai, China). SODs, CATs, and MDA assay kits were purchased from Nanjing Jiancheng Bioengineering Institute (Nanjing, China). RNAeasy™ Plus, PrimeScript™ RT reagent Kit, and TB Green^®^ Premix Ex Taq™ II (Tli RNaseH Plus) were purchased from Takara.

### 2.2. C. elegans Strains and Maintenance

All *C. elegans* strains were obtained from Caenorhabditis Genetics Centre (CGC), and cultivated at 20 °C on nematode growth medium (NGM). Strains used in the study were as following: wild-type N2; TJ356 *daf-16*p::GFP (zls356) IV.; TJ375 *hsp-16.2*p::GFP (gpls1); CF1553 *sod-3*p::GFP (muls84); CB1370 *daf-2* (e1370) III.; CF1038 *daf-16* (mu86) I.; TJ1052 *age-1* (hx546) II.; VC204 *akt-2* (ok393) X.; VC345 *sgk-1* (ok538) X.; EU1 *skn-1* (zu67) IV. Synchronized progeny was obtained as described previously [10].

### 2.3. Lifespan Assay

Synchronized L1 larvae were cultured on NGM with or without OLE or RES. Worms were transferred to the fresh NGM plates every day, then recorded all the alive, escaped, and dead data. Nematodes were scored as dead when they did not respond to a mechanical stimulus with a platinum wire.

### 2.4. Body Length and Width Measurements

Synchronized L1 larvae were maintained on NGM with or without OLE or RES for 48 h. Subsequently, 50 worms were transferred on the microscope slide with 1% sodium azide. The pictures were recorded by a microscope (Olympus BX53, Tokyo, Japan) under the bright field. The body length and width of the worm were measured via Image J software.

### 2.5. Heat Stress Assay

About 50 synchronized L1 larvae were transferred on NGM with or without OLE or RES. After 48 h, worms were moved to an incubator at 35 °C. The alive, escaped, and dead worms were checked for 12 h continuously at 2 h intervals. The results were given as the percentage of survival nematodes. The assays were carried out in triplicate with about 150 nematodes per group. The statistic analysis was performed by a log-rank (Mantel-Cox) statistical test.

### 2.6. Oxidative Stress Assay

Oxidative stress assay was performed as reported previously with some modifications [8]. About 50 synchronized L1 larvae were treated with or without OLE or RES. After 48 h, worms were exposed to 200 µM of paraquat. The alive, escaped, and dead worms were checked for 12 h continuously at 2 h intervals. The results were given as the percentage of survival nematodes. The assays were carried out in triplicate with about 150 nematodes per group. The statistic analysis was performed by a log-rank (Mantel-Cox) statistical test.

### 2.7. Measurement of Malondialdehyde (MDA) Content and Antioxidant Enzyme Activities

Approximately 4000 synchronized L1 larvae were cultivated with or without OLE or RES. After 48 h, worms were exposed to 35 °C for 3 h to induce activities of SODs and CATs. Then the worms were harvested by M9 buffer and crushed by an ultrasonic wave. The suspension was collected and centrifuged at 2500 rpm/min for 10 min. The MDA content and antioxidant enzyme activities in the supernatant were measured using respective assay kits (Nanjing Jiancheng Bioengineering Institute, Nanjing, China). The tests were repeated in triplicate.

### 2.8. Quantification sod-3p::GFP Expression

Synchronized transgenic strain CF1553 worms were cultured with or without OLE or RES from the L1 larva. After 48 h, the fluorescence of worms was measured from a microscope (BX53, Olympus Corporation, Tokyo, Japan). At least 25 worms per group were used to analyze the fluorescence intensity of *sod-3*p::GFP. The tests were repeated in triplicate.

### 2.9. Quantification hsp-16.2p::GFP Expression

Synchronized transgenic strain TJ375 worms were cultured with or without OLE or RES from the L1 larva. After 48 h, mild heat stress (35 °C for 20 min) was applied to assist the expression of *hsp-16.2*p::GFP. The fluorescence of worms was examined by a laser scanning confocal microscope (FV1200, Olympus Corporation, Tokyo, Japan). At least 25 worms per group were used to quantify the fluorescence intensity of *hsp-16.2*p::GFP. The tests were repeated in triplicate.

### 2.10. DAF-16::GFP Localization Assay

Synchronized transgenic strain TJ356 worms were cultured with or without OLE or RES for 48 h. TJ356 worms were monitored DAF-16::GFP signal by a laser scanning confocal microscope (FV1200, Olympus Corporation, Tokyo, Japan). At least 25 worms per group were used to analyze the subcellular DAF-16 distribution. According to the DAF-16::GFP distribution pattern, TJ356 worms were classified into three groups: worms with diffused fluorescence throughout the whole body were recorded as “cytosolic”; worms with both diffused and punctate fluorescence were recorded as “intermediate”; worms with punctate fluorescence were recorded as “nuclear”. The tests were repeated in triplicate.

### 2.11. Analysis of Genes Expression by qRT-PCR

Total RNA was extracted from about 2000 synchronized L1 larvae with or without compounds treatment for 48 h using RNAeasy™ Plus and then converted to cDNA using PrimeScript™ RT reagent Kit with gDNA Erase. The qRT-PCR was performed using TB Green^®^ Premix Ex Taq™ II (Tli RNaseH Plus) on the BioRad CFX96 system (BioRad, Hercules, CA, USA). Genes and primers used here are listed in Table 1.

### 2.12. Statistical Analysis

Survival analysis was performed by GraphPad Prism software, and data were expressed as means ± SEM of triplicate of assays. Statistical significance analysis was calculated by using ANOVA with Tukey’s post-hoc test. * means that *p*-value compared to control group. # means that *p*-value compared to RES group. * or # represents *p <* 0.05, ** or ## represents *p <* 0.01, *** or ### represents *p <* 0.001. Letter “ns” means no significance.

## 3. Results

### 3.1. OLE Extends C. elegans Lifespan

To evaluate if OLE could prolong the lifespan, the wild-type N2 worms were treated with a series of concentrations of OLE. The results showed that OLE extended the lifespan of *C. elegans* in a dose-dependent mode. A total of 500 µM RES as a positive control could significantly prolong the mean lifespan of N2 by 30.19% (*p <* 0.0001), while 440 µM OLE extended the mean lifespan of N2 by 22.30% (*p <* 0.0001), compared to the control group (Figure 1b and Table S1). Coincidentally, a previous study reported that OLE exhibited anti-aging activity in human embryonic fibroblasts by increasing the lifespan by 15.0% [11]. Considered highly homologous of genes between worms and humans, we could summarize that OLE extends the lifespan of different organisms through a conserved mechanism.

**Figure 2:**
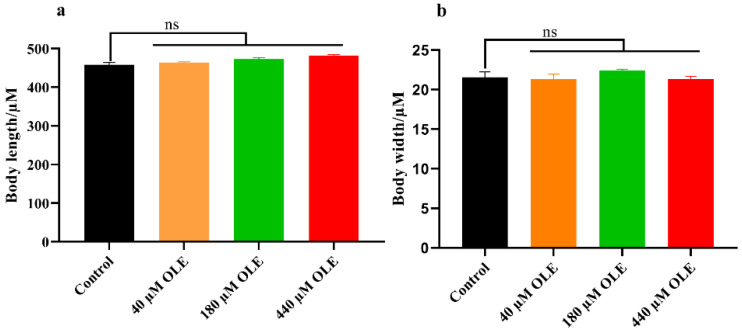
Effect of OLE on the body size of *C. elegans*. (**a**) Body length; (**b**) body width.

The pro-longevity effect of compounds on *C. elegans* is often accompanied by the change of body size. Therefore, the body length and width were measured after OLE treatment. As shown in Figure 2, OLE has no significant effect on the body length and width of *C. elegans* in comparison to the control group. These findings indicated that the lifespan extension of OLE under selected concentration might not be a result of arrested development or restricted calorie intake.

### 3.2. OLE Enhances Resistance of C. elegans against Heat and Oxidative Stress

**Figure 3:**
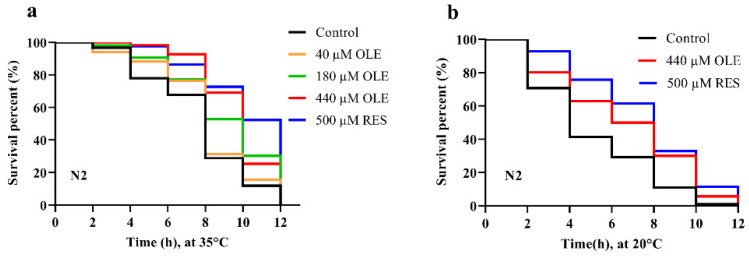
OLE increased the mean lifespan of N2 worms under (**a**) heat and (**b**) oxidative stress.

The lifespan extension effect is highly correlated with the increased environmental stress resistance in *C. elegans* [12,13,14]. In this study, after pretreatment of OLE for 48 h, the worms were exposed to 35 °C to induce heat shock. As shown in Figure 3a, 180 and 440 µM OLE significantly increased the mean lifespan of N2 worms by 24.83% (*p <* 0.001) and 35.33% (*p <* 0.0001), compared to control group (Table S2). Thus, the 440 µM OLE was selected for subsequent assays. Thermal stress also induces oxidative stress by generating intracellular ROS. To evaluate the oxidative stress resistance effect induced by OLE treatment, the worms were treated with 440 µM OLE for 48 h. After exposure to 200 µM paraquat to induce acute oxidative stress, 440 µM OLE was shown to extend the mean lifespan of N2 worms by 29.72% (*p <* 0.05), compared to the control group (Figure 3b and Table S2).

### 3.3. OLE Reduces the MDA Content and Enhances Antioxidant Enzymes Activities

**Figure 4:**
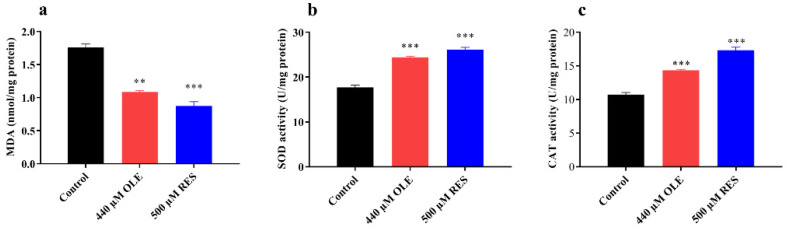
Effect of OLE on MDA content and antioxidant enzyme activities. (**a**) OLE reduced MDA contents; (**b**) OLE increased SODs activities; (**c**) OLE increased the CATs activities. ** *p* < 0.01 and *** *p* < 0.001 suggests significant difference when compared to control group.

MDA is the majority product of lipid peroxidation [15]. The content of MDA is generally used as a marker to evaluate the oxidative damage in organisms during aging. In this test, OLE could reduce MDA content by 38.43% (*p <* 0.01) (Figure 4a). Meanwhile, since OLE significantly increased the survival rate against oxidative and heat stress in worms, the activity of antioxidant enzymes, superoxidase dismutases (SODs), and catalases (CATs) was studied. As shown in Figure 4b,c, 48 h pretreatment of OLE increased the SODs and CATs activities by 38.00% (*p <* 0.001) and 33.84% (*p <* 0.001), respectively, compared to control group. Mendler showed that the overexpression of SODs and CATs could increase organism lifespan and antioxidant activities [16]. These results suggest that OLE might activate antioxidant enzymes activities to decrease stress damage and lipid peroxidation, then further prolong healthspan.

### 3.4. OLE Increases Expression of sod-3p::GFP in C. elegans

**Figure 5:**
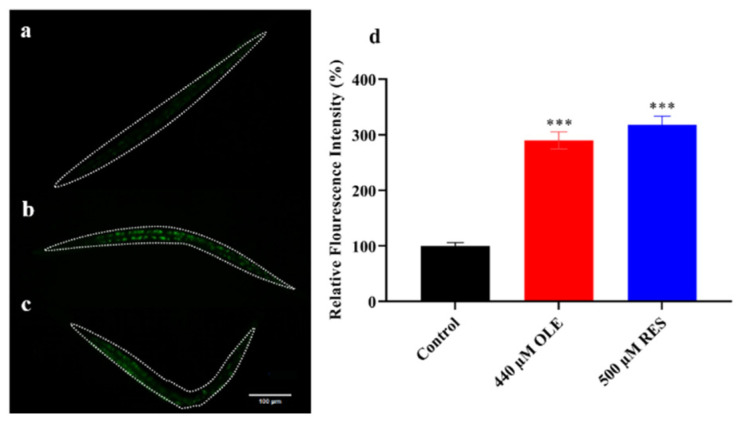
Effect of OLE on *sod-3*p::GFP expression. (**a**) Control group; (**b**) OLE treatment group; (**c**) RES treatment group; (**d**) OLE and RES increased the expression of *sod-3*p::GFP. Data are presented as relative GFP intensity compared to the control. *** *p* < 0.001 suggests a significant difference when compared to the control group.

**Figure 6:**
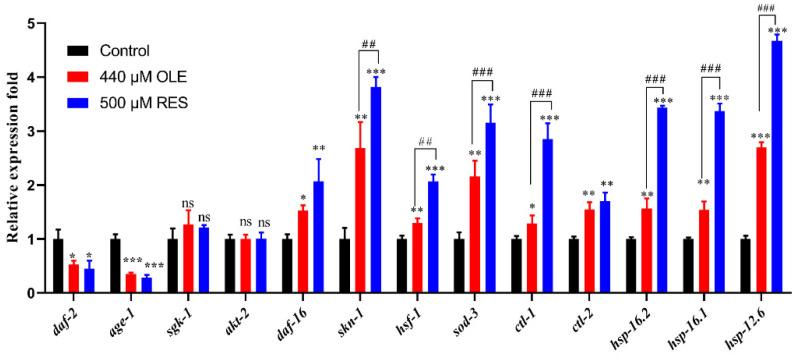
Effect of OLE on gene expression of N2 worms. Gene transcription level normalized to internal control gene *act-1*. * *p* < 0.05, ** *p* < 0.01, and *** *p* < 0.001 suggests a significant difference when compared to the control group, while *## p* < 0.01 and *### p* < 0.001 suggests a significant difference when compared OLE with RES group, ns: no significance.

Antioxidant enzymes, such as superoxidase dismutases and catalases, play a vital role in the free radical-scavenging system in organisms. SODs convert O_2_^•−^ into H_2_O_2_, and CATs catalyze the H_2_O_2_ into H_2_O and O_2_ [9]. In *C. elegans*, DAF-16 downstream targeted genes, *sod-3*, *ctl-1,* and *ctl-2,* encode antioxidant enzyme members of SOD-3, CTL-1, and CTL-2, respectively [9,17]. To examine whether OLE also induced the expression of antioxidant enzyme genes, the expression intensity of *sod-3*p::GFP in the CF1553 strain with OLE treatment was measured. The genetically modified strain of CF1553 carried the GFP reporter, which was activated by a *sod-3* promoter. As shown in Figure 5, OLE could increase the *sod-3*::GFP expression by 2.90-fold (*p <* 0.001) compared to the control group. Moreover, in relative quantification of genes expression test, as compared to the control group, the quantified mRNA levels of antioxidant enzyme genes showed that OLE could up-regulate *sod-3* and *ctl-2* expression by 2.16-fold (*p <* 0.01) and 1.55-fold (*p <* 0.01), respectively, while OLE up-regulate *ctl-1* expression by 1.29-fold (*p <* 0.05) (Figure 6). The different effects of OLE on *ctl-1* and *ctl-2* might be because CTL-2 contributes up to 80% of the total CATs activity in *C. elegans*. These results suggest that OLE significantly induced the expression of stress resistance-related genes, which was in line with the lifespan extension of *C. elegans* under normal growth and harsh environmental conditions (Figure 3).

### 3.5. OLE Strengthens the Expression of hsp-16.2p::GFP in C. elegans

**Figure 7:**
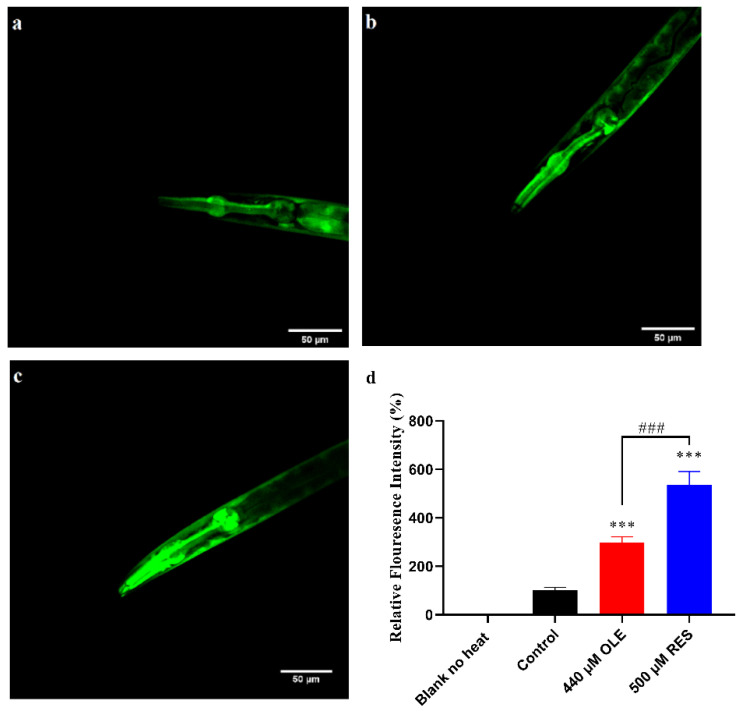
Effect of OLE on the expression of *hsp-16.2*p::GFP under heat stress. (**a**) Control group; (**b**) OLE treatment group; (**c**) RES treatment group; (**d**) OLE significantly increased the expression of *hsp-16.2*p::GFP. Data are presented as relative GFP intensity compared to the control. *** *p* < 0.001 suggests a significant difference when compared to the control group, while *### p* < 0.001 suggests a significant difference when compared OLE with the RES group.

OLE treatment significantly increased the heat stress tolerance in vivo. In worms, the heat stress response is mainly regulated by a conservative chaperones family, small heat shock proteins (sHSPs). In this study, the mutant TJ375 was used to evaluate the effect of OLE on HSP-16.2, an sHSP member distributed throughout the worm body. As shown in Figure 7, there is no measurable fluorescence in the blank group without any treatment. After 20 min mild heat stress, pretreatment with OLE and RES significantly enhanced the expression of *hsp-16.2*p::GFP by (*p <* 0.001) and 5.36-fold (*p <* 0.001), respectively, compared to the control group. Similar results were shown in mRNA levels of sHSPs genes, which OLE remarkably induced the genes expression of *hsp-16.1*, *hsp-16.2* and *hsp-12.6* by 1.40-fold (*p <* 0.01), 1.57-fold (*p <* 0.01), and 2.70-fold (*p <* 0.001), respectively (Figure 6).

### 3.6. OLE Promotes Nuclear Translocation of DAF-16

**Figure 8:**
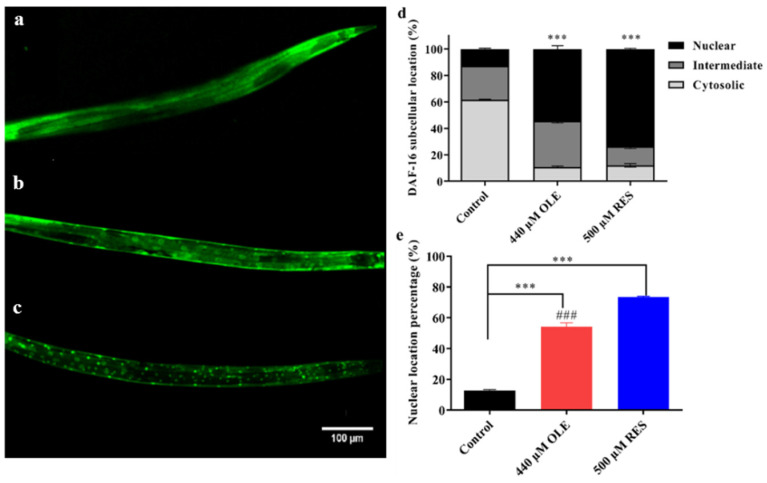
Effect of OLE on the subcellular localization of DAF-16::GFP. (**a**) Cytosolic translocation; (**b**) Intermediate translocation; (**c**) Nuclear translocation; (**d**) Influence of OLE on the localization of DAF-16::GFP. The histogram represents the fraction of TJ356 worms number with cytosolic, intermediate, or nuclear localization of DAF-16::GFP; (**e**) OLE significantly induced the nuclear translocation of DAF-16::GFP. *** *p* < 0.001 suggests a significant difference when compared to the control group, while *### p* < 0.001 suggests a significant difference when compared OLE with the RES group.

In worms, the expression of antioxidant enzyme genes, such as *sod-3*, *ctl-1*, and *ctl-2*, and the expression of sHSPs genes, such as *hsp-16.1*, *hsp-16.2,* and *hsp-12.6*, are mainly modulated by a FoxO transcription factor, DAF-16. DAF-16/FoxO was shown to improve stress resistance and promote longevity across species [18]. Under normal growth conditions, DAF-16 is mostly distributed in the cytoplasm (Figure 8a). However, under stress, DAF-16 will gradually transfer from the cytoplasm to the nucleus (Figure 8b,c) and further promote the transcription of downstream genes to improve the stress resistance or extend the lifespan of *C. elegans*.

As shown in Figure 8e, approximately 12.77% of worms exhibited nuclear localization of DAF-16:GFP in control groups. While pretreatment with OLE and RES significantly induced the translocalization of DAF-16::GFP from the cytoplasm to the nucleus, which increased the percentage to 54.32% and 73.52%, respectively. The nuclear localization of DAF-16 is most likely to induce stress response-related genes, such as *sod-3*, *ctl-1/2*, *hsp-12.6*, *hsp-16.1, and hsp-16.2,* to prolong the healthspan. Similar results were revealed by many natural compounds, such as Tyrosol [19], carnosic acid [20], and Gengnianchun [21].

### 3.7. OLE Extends Healthspan of C. elegans via IIS Pathway and SKN-1/Nrf2 Transcription Factor

**Figure 9:**
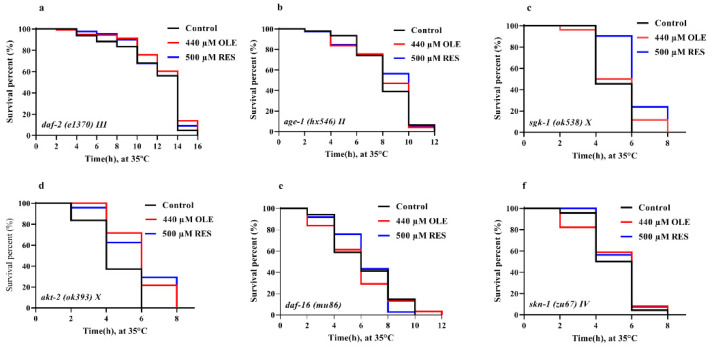
Effect of OLE on healthspan of worms under heat stress (**a**) *daf-2* mutant worms; (**b**) *age-1* mutant worms; (**c**) *sgk-1* mutant worms; (**d**) *akt-2* mutant worms; (**e**) *daf-16* mutant worms; (**f**) *skn-1* mutant worms.

The mechanism of action that IIS regulates DAF-16 to prolong lifespan is well characterized by many studies. Briefly, suppression of the insulin-like signaling cascade activates the nuclear translocation of DAF-16, resulting in the amelioration effects on healthspan. Components in the IIS pathway, *age-1*, *akt-1/2*, *sgk-1,* and *daf-2,* are all involved in the cascade to regulate the activity of transcription factor DAF-16. To investigate if OLE supplementation mediated the lifespan in an IIS-dependent manner, the mRNA expression of these IIS components was evaluated. As shown in Figure 6, OLE significantly down-regulated the expression of *daf-2* and *age-1* by 0.53-fold (*p <* 0.05) and 0.35-fold (*p <* 0.001), respectively. The same trend was found in positive control group of RES, decreasing with 0.45-fold (*p <* 0.05) and 0.29-fold (*p <* 0.001), respectively. Neither OLE nor RES had significant effects on *akt-2* and *sgk-1*, but both of them could up-regulate the expression of *daf-16* by 1.53-fold (*p <* 0.05) and 2.07-fold (*p <* 0.001). These results suggest that OLE might inhibit the upstream gene expression of the IIS pathway, including *daf-2* and *age-1*, then enhance the *daf-16* expression to mediate genes related to stress resistance and longevity. To further confirm whether OLE mediated the healthspan via the IIS pathway, the beneficial effect of OLE on the IIS pathway-related null mutants was investigated. As shown in Figure 9 and Table S3, OLE did not affect the lifespan of *daf-2*, *age-1*, *sgk-1,* and *daf-16* null mutants compared to the control group. These results were in accord with mentioned mRNA data, indicating that OLE extended the lifespan of *C. elegans* via the IIS pathway.

In a recent study, OLE was reported to attenuate oxidative stress by activating the Nrf2 pathway [22]. Therefore, whether Nrf2 is involved in OLE-induced longevity effect was also investigated. In *C. elegans*, the *skn-1* gene encoded a conserved Nrf2 transcription factor. SKN-1/Nrf2 can interact with other co-factors, such as DAF-16/FoxO, to enhance various stress responses, including heat, oxidative stress, etc. [23,24]. Thus, whether OLE-mediated healthy benefits required *skn-1* was also investigated. The results showed that OLE could increase the *skn-1* expression by 2.69-fold (*p* < 0.01) compared to the control group and failed to prolong the mean lifespan of the *skn-1* null mutant (Figure 6 and Figure 9 and Table S3). The findings suggest that OLE prolonged the lifespan through the SKN-1/Nrf2 transcription factor.

## 4. Discussion

Aging represents a progressive degeneration of physiological functions and metabolic processes. It is the main risk factor of several chronic disorders, including most neurodegenerative diseases. Accumulating evidence has demonstrated that drug interference, in particular bioactive natural compounds, could regulate longevity signal pathways to extend median lifespan and maintain healthspan in living organisms [25,26,27]. OLE is the major bioactive compound present in olive fruit and leaves. The previous studies showed that OLE exhibited some anti-aging activities in the cell model [11] and decreased Aß aggregation, which is inherent to aging-related Alzheimer’s disease [28]. However, the exact effect of OLE on healthspan in organisms is still largely uncharacterized. In the present study, OLE extended the lifespan of *C. elegans* in a dose-dependent mode. The mean lifespan of wild-type nematode was maximally increased by 22.30% in the treatment group of 440 µM OLE. During aging, the body augments vulnerability to external and internal stress, leading to a number of debilitating diseases and an increase in mortality. Under paraquat-induced oxidative stress and heat stress, the decreased antioxidant enzymes activities or an increase in reactive oxygen species would break the redox balance, resulting in oxidative damage of living organisms. Longevity extension and stress resistance accompany the downturn of oxidative damage. In this study, OLE significantly stimulated antioxidant enzymes activities, reduced MDA content (Figure 4), further alleviated oxidative damage, and promoted the healthspan. Numerous studies also showed that phytochemicals enhanced resistance against stress contributes to longevity [29]. For example, carnosol could ameliorate the health span of *C. elegans* via increasing antioxidant enzyme activities and reducing MDA content [26].

The IIS pathway plays a critical role in regulating longevity and resistance stress via a conserved mechanism. Reduction in the IIS pathway contributes to the activation of longevity transcription factor DAF-16, which mediates genes involved in intrinsic antioxidant enzymes system, such as SODs and CATs [30]. OLE could promote approximate 54.32% of DAF-16 to transfer from the cytoplasm to the nucleus (Figure 8), thereby up-regulating the expression of *sod-3*, *ctl-1,* and *ctl-2* (Figure 5 and Figure 6), which were consistent with the results of enhanced stress resistance (Figure 3) and antioxidant enzymes activities in worms (Figure 4). Insulin-like peptides receptor, DAF-2, and its downstream kinase cascade, including AGE-1, AKT-1/2, and SGK-1, are the key components involved in the IIS longevity pathway of *C. elegans*. The inhibition of DAF-2 leads to the inactivation of AGE-1/PI3K. In turn, the decreased function of AGE-1 results in the inactivation of the downstream kinases, including the inactivation of PDK-1, AKT-1/2, and SGK-1. In addition, inactivated AKT-1/2 and SGK-1 suppress the phosphorylation of DAF-16/FoxO, inducing its nuclear translocation to activate the longevity genes. In this study, OLE significantly inhibited the expression of *daf-2* and *age-1* but had no effect on the expression of *akt-2* and *sgk-1*. These gene expression data suggest that *akt-2* might not be included in the OLE-mediated IIS pathway, which was in accordance with the result that the increased survival rate by OLE treatment was not eliminated by *akt-2* knockout. IIS transduction might occur via either its isoform *akt-2* or other components paralleling to AKTs, such as *sgk-1*. Although OLE could not affect the expression of *sgk-1* as well, the corresponding null mutant study, OLE could not extend the lifespan of *sgk-1* deficiency strain, suggesting that *sgk-1* might involve in OLE-induced effects. These results indicate that OLE supplementation partially suppressed the IIS pathway in worms.

As a result of inhibited IIS, the downstream of IIS, *daf-16*, *sod-3*, *ctl-1,* and *ctl-2* was remarkably induced (Figure 6), which were in suitable agreement with the findings of promoting nuclear translocation of DAF-16 and increased antioxidant enzymes activities (Figure 4, Figure 5 and Figure 8). Additionally, OLE could not further extend the mean lifespan of *daf-2*, *age-1*, *sgk-1,* and *daf-16* loss-function mutants, suggesting that the lifespan extension effect of OLE on *C. elegans* required the IIS pathway. Another output of the IIS-regulated longevity pathway, HSF-1, is a heat-shock transcription factor that induces small heat shock proteins (sHSPs) in response to heat and oxidative stress. In this study, OLE also increased the expression of *hsf-1*, *hsp-16.1*, *hsp-16.2,* and *hsp-12.6*, and enhanced the *hsp-16.2*p::GFP under heat stress, suggesting the HSF-1 and sHSPs might also be involved in OLE-induced longevity effect and stress resistance.

In addition to DAF-16, the reduction in the IIS pathway also leads to the activation of longevity-promoting transcription factors SKN-1/Nrf2. SKN-1/Nrf2 is an oxidative stress-responsive transcription factor that mediates stress responses and lifespan through numerous genes [31], many of which overlap with DAF-16. In this study, OLE could appreciably up-regulate the expression of *skn-1* (Figure 6) but could not extend the mean lifespan of *skn-1* loss-function-mutant, indicating that the lifespan extension effect of OLE also demand SKN-1. Similar results were undertaken by Duangjan et al., showing that Glochidion zeylanicum leaf extracts extended the healthspan of *C. elegans* depending on the SKN-1/Nrf2 transcription factor [9].

## 5. Conclusions

The present results showed that OLE could protect worms from heat shock and paraquat stress. Supplementation with OLE significantly increased the lifespan of wild-type N2 worms. OLE treatment promoted the migration of DAF-16 into the nucleus, which induced the activities and expressions of antioxidant enzymes, including *sod-3*, *ctl-1*, and *ctl-2*. The lipid peroxidation products MDA was reduced by OLE treatment as well. Simultaneously, treatment with OLE up-regulated the mRNA contents of *daf-16*, *skn-1*, *hsf-1*, *hsp-16.1*, *hsp-16.2*, and *hsp-12.6*, while down-regulated the expressions of *daf-2* and *age-1*. In addition, the null mutation in genes of *daf-2*, *akt-2*, *sgk-1*, *daf-16*, and *skn-1* abolished the lifespan-extending effect of OLE. These results suggest that OLE enhanced the stress resistance and prolonged the lifespan of worms via the IIS pathway and SKN-1/Nrf2 transcription factor. The present study demonstrates the healthspan promoting effect of OLE and provides a potential mechanistic explanation for OLE against aging and aging-related decline, which might contribute to the exploitation of nutraceuticals from olives. Further studies are needed to explore other longevity pathways mediated by OLE and clarify the therapeutic benefits in more complex model animals.

## Figures and Tables

**Table 1:** Primer sequences of genes used in the experiment.

Gene Accession Number	Gene	Primer
Gene ID: 179535	*act-1* F	5’-CCAGGAATTGCTGATCGTATGCAGAA-3’
Gene ID: 179535	*act-1* R	5’-TGGAGAGGGAAGCGAGGATAGA-3’
Gene ID: 175410	*daf-2* F	5’-GGATAAAGGCGAATCAAAGTGTC-3’
Gene ID: 175410	*daf-2* R	5’-CGATACACTTTCCCTTGTGATAGAC-3’
Gene ID: 174762	*age-1* F	5’-CCTGAACCGACTGCCAATC-3’
Gene ID: 174762	*age-1* R	5’-GTGCTTGACGAGATATGTGTATTG-3’
Gene ID: 181524	*akt-2* F	5′-ACATTCAGCGAAGCACGAACA-3′
Gene ID: 181524	*akt-2* R	5′-TACATGACCACTCCGACTCCC-3′
Gene ID: 172981	*daf-16* F	5′-TCGTCGTCTCGTGTTTCTCCA-3′
Gene ID: 172981	*daf-16* R	5′-TTCCATAGGCACCCGGTAGTG-3′
Gene ID: 181697	*sgk-1* F	5′-CACCGACTTTGGGCTCTGTAA-3′
Gene ID: 181697	*sgk-1* R	5′-CTTGAGACGAAGTGGCTGGTT-3′
Gene ID: 177343	*skn-1* F	5′-AGTGTCGGCGTTCCAGATTTC-3′
Gene ID: 177343	*skn-1* R	5′-GTCGACGAATCTTGCGAATCA-3′
Gene ID: 173078	*hsf-1* F	5′-TTGACGACGACAAGCTTCCAGT-3′
Gene ID: 173078	*hsf-1* R	5′-AAAGCTTGCACCAGAATCATCCC-3′
Gene ID: 181748	*sod-3* F	5′-CTAAGGATGGTGGAGAACCTTCA-3′
Gene ID: 181748	*sod-3* R	5′-CGCGCTTAATAGTGTCCATCAG-3′
Gene ID: 178659	*hsp-16.2* F	5′-CTGCAGAATCTCTCCATCTGAGTC-3′
Gene ID: 178659	*hsp-16.2* R	5′-AGATTCGAAGCAACTGCACC-3′
Gene ID: 259738	*ctl-1* F	5′-TTTCAACGGTCGCTGGAGAA-3′
Gene ID: 259738	*ctl-1* R	5′-AGTCTGTGGATTGCGCTTCA-3′
Gene ID: 175085	*ctl-2* F	5′-TTCGCTGAGGTTGAACAATCCG-3′
Gene ID: 175085	*ctl-2* R	5′-GTTGCTGATTGTCATAAGCCATTGC-3′
Gene ID: 179286	*hsp-16.1* F	5′-GTCACTTTACCACTATTTCCGTCCAGCTCAACGTTC-3′
Gene ID: 179286	*hsp-16.1* R	5′-CAACGGGCGCTTGCTGAATTGGAATAGATCTTCC-3′
Gene ID: 177778	*hsp-12.6* F	5′-GTGATGGCTGACGAAGGAAC-3′
Gene ID: 177778	*hsp-12.6* R	5′-GGGAGGAAGTTATGGGCTTC-3′

## Data Availability

Data is contained within the article or Supplementary Materials.

## Supplementary Materials

The following are available online at https://www.mdpi.com/article/10.3390/antiox10111697/s1, Table S1. Lifespan summary statistics, Table S2. Heat and oxidative stress summary statistics, Table S3. Lifespan summary statistics under heat stress.

## References

[1] Hayes J., Allen P., Brunton N., O’Grady M., Kerry J. (2011). Phenolic composition and in vitro antioxidant capacity of four commercial phytochemical products: Olive leaf extract (*Olea europaea* L.), lutein, sesamol and ellagic acid. Food Chem..

[2] Nikolaivits E., Termentzi A., Skaltsounis A.-L., Fokialakis N., Topakas E. (2017). Enzymatic tailoring of oleuropein from Olea europaea leaves and product identification by HRMS/MS spectrometry. J. Biotechnol..

[3] Omar S.H. (2010). Oleuropein in olive and its pharmacological effects. Sci. Pharm..

[4] Menendez J.A., Joven J., Aragonès G., Barrajón-Catalán E., Beltrán-Debón R., Borrás-Linares I., Camps J., Corominas-Faja B., Cufí S., Fernández-Arroyo S. (2013). Xenohormetic and anti-aging activity of secoiridoid polyphenols present in extra virgin olive oil. Cell Cycle.

[5] Rigacci S., Vassallo N. (2015). Olive Oil Phenols as Promising Multi-targeting Agents against Alzheimer’s Disease. Natural Compounds as Therapeutic Agents for Amyloidogenic Diseases.

[6] Maryam S., Fereshteh M., Mansooreh S. (2014). Antioxidant role of oleuropein on midbrain and dopaminergic neurons of substantia nigra in aged rats oleuropein ameliorates arsenic induced oxidative stress in mice. Iran. Biomed. J..

[7] Metin O., Ayla O., Musa K., Oguz M., Hasan O., Abdulsamed K., Mahmut K. (2016). Oleuropein ameliorates arsenic induced oxidative stress in mice. J. Trace Elem. Med. Bio..

[8] Daitoku H., Fukamizu A. (2007). FOXO transcription factors in the regulatory networks of longevity. J. Biochem..

[9] Duangjan C., Rangsinth P., Gu X., Zhang S., Wink M., Tencomnao T. (2019). Glochidion zeylanicum leaf extracts exhibit lifespan extending and oxidative stress resistance properties in *Caenorhabditis elegans* via DAF-16/FoxO and SKN-1/Nrf-2 signaling pathways. Phytomedicine.

[10] Feng S., Cheng H., Xu Z., Yuan M., Huang Y., Liao J., Yang R., Zhou L., Ding C. (2018). Panax notoginseng polysaccharide increases stress resistance and extends lifespan in *Caenorhabditis elegans*. J. Funct. Foods.

[11] Katsiki M., Chondrogianni N., Chinou I., Rivett A.J., Gonos E.S. (2007). The Olive Constituent Oleuropein Exhibits Proteasome Stimulatory Properties In Vitro and Confers Life Span Extension of Human Embryonic Fibroblasts. Rejuv. Res..

[12] Benedetti M.G., Foster A.L., Vantipalli M.C., White M.P., Sampayo J.N., Gill M.S., Olsen A., Lithgow G.J. (2008). Compounds that confer thermal stress resistance and extended lifespan. Exp. Gerontol..

[13] Finkel T., Holbrook N.J. (2000). Oxidants, oxidative stress and the biology of ageing. Nature.

[14] Lithgow G.J., Walker G.A. (2002). Stress resistance as a determinate of *C. elegans* lifespan. Mech. Ageing Dev..

[15] Schieber M., Chandel N.S. (2014). ROS function in redox signaling and oxidative stress. Curr. Biol..

[16] Mendler M., Riedinger C., Schlotterer A., Volk N., Fleming T., Herzig S., Nawroth P.P., Morcos M. (2016). Reduction in ins-7 gene expression in non-neuronal cells of high glucose exposed *Caenorhabditis elegans* protects from reactive metabolites, preserves neuronal structure and head motility, and prolongs lifespan. J. Diabetes Its Complicat..

[17] Suthammarak W., Somerlot B.H., Opheim E., Sedensky M., Morgan P.G. (2013). Novel interactions between mitochondrial superoxide dismutases and the electron transport chain. Aging Cell.

[18] McElwee J., Bubb K., Thomas J.H. (2003). Transcriptional outputs of the *Caenorhabditis elegans* forkhead protein DAF-16. Aging Cell.

[19] Cañuelo A., Gilbert-Lopez B., Liñán P.J.P., Martínez-Lara E., Siles E., Miranda-Vizuete A. (2012). Tyrosol, a main phenol present in extra virgin olive oil, increases lifespan and stress resistance in *Caenorhabditis elegans*. Mech. Ageing Dev..

[20] Lin C., Zhang X., Xiao J., Zhong Q., Kuang Y., Cao Y., Chen Y. (2019). Effects on longevity extension and mechanism of action of carnosic acid in *Caenorhabditis elegans*. Food Funct..

[21] Meng F., Li J., Rao Y., Wang W., Fu Y. (2018). Gengnianchun Extends the Lifespan of *Caenorhabditis elegans* via the Insulin/IGF-1 Signalling Pathway. Oxidative Med. Cell. Longev..

[22] Sun W., Wang X., Hou C., Yang L., Li H., Guo J., Huo C., Wang M., Miao Y., Liu J. (2017). Oleuropein improves mitochondrial function to attenuate oxidative stress by activating the Nrf2 pathway in the hypothalamic paraventricular nucleus of spontaneously hypertensive rats. Neuropharmacology.

[23] An J.H., Vranas K., Lucke M., Inoue H., Hisamoto N., Matsumoto K., Blackwell T.K. (2005). Regulation of the *Caenorhabditis elegans* oxidative stress defense protein SKN-1 by glycogen synthase kinase-3. Proc. Natl. Acad. Sci. USA.

[24] Tullet J.M., Hertweck M., An J.H., Baker J., Hwang J.Y., Liu S., Oliveira R.P., Baumeister R., Blackwell T.K. (2008). Direct Inhibition of the Longevity-Promoting Factor SKN-1 by Insulin-like Signaling in *C. elegans*. Cell.

[25] Carranza A.D.V., Saragusti A., Chiabrando G.A., Carrari F., Asis R. (2020). Effects of chlorogenic acid on thermal stress tolerance in *C. elegans* via HIF-1, HSF-1 and autophagy. Phytomedicine.

[26] Lin C., Zhang X., Su Z., Xiao J., Lv M., Cao Y., Chen Y. (2019). Carnosol improved lifespan and healthspan by promoting antioxidant capacity in *Caenorhabditis elegans*. Oxidative Med. Cell. Longev..

[27] Yang Z.-Z., Yu Y.-T., Lin H.-R., Liao D.-C., Cui X.-H., Wang H.-B. (2018). Lonicera japonica extends lifespan and healthspan in *Caenorhabditis elegans*. Free. Radic. Biol. Med..

[28] Omar S.H. (2010). Cardioprotective and neuroprotective roles of oleuropein in olive. Saudi Pharm. J..

[29] Ding A.-J., Zheng S., Huang X.-B., Xing T.-K., Wu G.-S., Sun H.-Y., Qi S.-H., Luo H.-R. (2017). Current perspective in the discovery of anti-aging agents from natural products. Nat. Prod. Bioprospect..

[30] Murphy C.T. (2013). Insulin/insulin-like growth factor signaling in *C. elegans*. WormBook.

[31] Blackwell T.K., Steinbaugh M.J., Hourihan J.M., Ewald C.Y., Isik M. (2015). SKN-1/Nrf, stress responses, and aging in *Caenorhabditis elegans*. Free Radic. Biol. Med..

