# Supplementary material for: Oleuropein Enhances Stress Resistance and Extends Lifespan via Insulin/IGF-1 and SKN-1/Nrf2 Signaling Pathway in Caenorhabditis elegans
Source: Antioxidants (Basel). 2021 Oct 27;10(11):1697. doi: 10.3390/antiox10111697 (PMC8614835; doi:10.3390/antiox10111697)
Supplement: Supplementary file 1 [file antioxidants-10-01697-s001.zip › antioxidants-1438138-supplementary.pdf]

Table S1. Lifespan summary statistics

Treatment	Mean Lifespan ± SEM (Day)	Number of Worms	*p*-Value vs. Control	*p*-Value vs. 500 μM RES	Lifespan Extension (%)
Control	15.07±0.736	153	-	<0.0001	-
40 μM OLE	15.98±0.523	157	0.0451	<0.0001	6.03%
180 μM OLE	17.57±0.349	155	<0.0001	0.0004	16.59%
440 μM OLE	18.43±0.332	161	<0.0001	0.0133	22.29%
500 μM RES	19.62±0.348	148	<0.0001	-	30.19%

The mean lifespan values were calculated by a log-rank (Mantel-Cox) statistical test.

Table S2. Heat and oxidative stress summary statistics

Condition	Treatment	Mean Lifespan ± SEM	Number of Worms	*p*-Value vs. Control	*p*-Value vs. 500 μM RES	Lifespan Change (%)
Heat stress	Control	7.53±0.165	154	-	<0.0001	-
	40 μM OLE	8.12±0.115	150	0.1065	<0.0001	7.84
	180 μM OLE	9.40±0.056	157	<0.001	<0.01	24.83
	440 μM OLE	10.19±0.034	162	<0.0001	0.0569	35.33
	500 μM RES	10.91±0.485	165	<0.0001	-	44.89
Oxidative stress	Control	5.08±0.312	158	-	-	-
	440 μM OLE	6.59±0.164	154	<0.05	0.1199	29.72
	500 μM RES	7.52±0.359	157	<0.01	-	48.03

The mean lifespan values were calculated by a log-rank (Mantel-Cox) statistical test.

Table S3. Lifespan summary statistics under heat stress

Genotype	Treatment	Mean Lifespan ± SEM (hour)	Number of Worms	*p*-Value vs. Control	*p*-Value vs. 500 μM RES
daf-2 (e1370) III	Control	11.65±0.264	159	-	-
	440 μM OLE	12.17±0.163	151	0.5640	0.6841
	500 μM RES	12.20±0.100	157	0.8607	-
age-1 (hx546) II	Control	7.76±0.312	163	-	-
	440 μM OLE	8.84±0.624	166	0.3030	0.2769
	500 μM RES	7.70±0.633	161	0.9470	-
sgk-1 (ok538) X	Control	4.91±0.210	155	-	-
	440 μM OLE	5.15±0.290	152	0.8295	0.0037
	500 μM RES	6.29±0.170	158	<0.01	-
akt-2 (ok393) X	Control	4.66±0.201	155	-	-
	440 μM OLE	5.76±0.275	167	<0.05	0.9118
	500 μM RES	5.72±0.094	154	<0.05	-
daf-16 (mu86)	Control	6.18±0.410	150	-	-
	440 μM OLE	5.81±0.470	159	0.6579	0.6210
	500 μM RES	6.27±0.330	154	0.7784	-
skn-1 (zu67) IV	Control	5.50±0.298	157	-	-
	440 μM OLE	5.27±0.408	160	0.6315	0.7623

500 μM RES	5.44±0.206	159	0.8567	-

The mean lifespan values were calculated by a log-rank (Mantel-Cox) statistical test.
